# Wenyang Jiedu Tongluo formula ameliorates diabetic kidney disease by regulating JAML/SIRT1 signaling to improve lipid metabolism in db/db mice

**DOI:** 10.3389/fphar.2025.1611585

**Published:** 2025-08-05

**Authors:** Yutong Liu, Tianying Chang, Zikun Wang, Hongkai Liu, Fan Li, Chengji Cui, Yingzi Cui, Shoulin Zhang

**Affiliations:** ^1^College of Traditional Chinese Medicine, Changchun University of Chinese Medicine, Changchun, China; ^2^Evidence-Based Office, The Affiliated Hospital to Changchun University of Chinese Medicine, Changchun, China; ^3^Nephropathy Department, The Affiliated Hospital to Changchun University of Chinese Medicine, Changchun, China; ^4^College of Integrated Chinese and Western Medicine, Changchun University of Chinese Medicine, Changchun, China

**Keywords:** diabetic kidney disease, lipid metabolism, Wenyang Jiedu Tongluo formula, JAML/SIRT1 signaling pathway, traditional Chinese medicine

## Abstract

**Introduction:**

Diabetic kidney disease (DKD), a major microvascular complication of diabetes mellitus, is closely associated with abnormal lipid metabolism, which contributes to secondary renal injury. The JAML/SIRT1 signaling pathway plays a critical role in regulating renal lipid metabolism during DKD progression. To investigate the molecular mechanisms underlying the therapeutic effects of Wenyang Jiedu Tongluo Formula (WYJDTLF) on lipid metabolism in DKD, we conducted an animal study using db/db mice.

**Methods:**

The mice were treated with WYJDTLF for 4 weeks, and its efficacy was evaluated through assessments of liver and kidney function, lipid profiles, and renal histopathology. Renal injury was examined using Hematoxylin and Eosin (H&E), Periodic Acid-Schiff (PAS), and Masson’s trichrome staining. Podocyte damage was assessed by quantifying the expression of podocyte marker proteins (Nephrin and NPHS2) using quantitative reverse transcription-polymerase chain reaction (qRT-PCR). Additionally, the expression levels of key proteins in the JAML/SIRT1 signaling pathway were analyzed via Western blot (WB).

**Results:**

The results demonstrated that WYJDTLF significantly improved liver and kidney function, reduced lipid deposition and inflammatory damage, and alleviated renal fibrosis and pathological injury. These effects were mediated through the regulation of the JAML/SIRT1 signaling pathway. Furthermore, WYJDTLF treatment upregulated the expression of Nephrin and NPHS2, indicating a protective effect on podocyte integrity.

**Conclusion:**

Our team has revealed for the first time that the WYJDTLF can improve lipid metabolism abnormalities in db/db mice and alleviate diabetic kidney disease-induced renal pathological damage by inhibiting the JAML/SIRT1 signalling pathway. These findings provide a scientific basis for the potential application of WYJDTLF in the treatment of DKD.

## 1 Introduction

Diabetic Kidney Disease (DKD), a significant microvascular complication of diabetes, poses a growing global health challenge. According to the IDF Global Diabetes Map 2021 (Sun et al., 2022) approximately half of individuals with type 2 diabetes mellitus (T2DM) and one-third of those with type 1 diabetes are at risk of developing chronic kidney disease (CKD) (Chen et al., 2025). The International Diabetes Foundation (IDF) reported in 2014 that diabetes accounts for 30%–47% of end-stage renal disease (ESRD) cases worldwide. Notably, the mortality rate of DKD patients undergoing dialysis is significantly higher than that of non-dialysis DKD patients (de Boer et al., 2011). Given these alarming trends, DKD has emerged as one of the most pressing international public health issues, underscoring the urgent need for effective prevention and treatment strategies (Lu et al., 2024).

DKD is clinically characterized by chronic persistent proteinuria, which serves as a key diagnostic marker. Pathologically, DKD is defined by uniform thickening of the glomerular basement membrane (GBM) and the development of glomerulosclerosis. These structural alterations disrupt normal renal hemodynamics, contributing to progressive renal dysfunction. Over time, these pathological changes significantly elevate the risk of end-stage renal disease (ESRD) (Lin et al., 2018; Reidy et al., 2014). The diagnosis of DKD is often supported by a long-standing history of diabetes mellitus, an elevated urinary albumin-to-creatinine ratio (UACR), and a reduced estimated glomerular filtration rate (eGFR). The pathogenesis of DKD is multifactorial, involving a complex interplay of mechanisms such as chronic hyperglycemia, excessive production of reactive oxygen species (ROS), oxidative stress, systemic inflammation, renal fibrosis, hemodynamic abnormalities, and dysregulation of cellular autophagy (Mazzieri et al., 2024). With the progressive elucidation of the pathogenesis of DKD, lipid metabolism has emerged as a critical area of focus. Dysphilia is recognized as an independent risk factor for DKD, contributing to the loss of vasoprotective, antioxidant, and anti-inflammatory properties. This metabolic disturbance induces lipotoxic injuries, including oxidative stress, inflammation, dysregulated autophagy, and apoptosis, which collectively exacerbate renal damage. Furthermore, dysphilia significantly increases the risk of cardiovascular and cerebrovascular diseases in DKD patients (Crociati et al., 2018; Fornoni and Merscher, 2020). Currently, the management of DKD includes general supportive therapy and pharmacological interventions such as angiotensin-converting enzyme inhibitors (ACEIs), angiotensin II receptor blockers (ARBs), sodium-glucose co-transporter-2 (SGLT-2) inhibitors, glucagon-like peptide-1 (GLP-1) receptor agonists, and dipeptidyl peptidase-4 (DPP-4) inhibitors. However, these therapeutic agents are associated with certain side effects and clinical limitations. For instance, ACEIs and ARBs may induce hyperkalemia, while SGLT-2 inhibitors are not recommended for patients with an eGFR below 15 mL/min/1.73 m^2^ and are contraindicated in those undergoing dialysis (Fujita et al., 2014; Groop et al., 2017).

Podocytes, a critical component of the glomerular filtration barrier, are highly susceptible to lipotoxicity. Ducasa et al. demonstrated that dysregulation of lipid metabolism leads to the accumulation of abnormal lipids and elevated intracellular free fatty acids. This lipotoxic environment induces irreversible damage to podocytes, compromising their structural and functional integrity, and this type of lipotoxicity leads to irreversible damage to podocytes (Ducasa et al., 2019). Emerging evidence suggests that the JAML/SIRT1 signaling pathway plays a pivotal role in regulating lipid metabolism processes. Activation of this pathway has been shown to mitigate abnormal lipid deposition and alleviate lipotoxicity-induced renal injury in patients with DKD (Gu et al., 2022). In patients with DKD, the expression levels of junctional adhesion molecule-like protein (JAML), sterol regulatory element-binding protein-1 (SREBP-1), and carbohydrate response element-binding protein (ChREBP) were found to be positively correlated with serum creatinine levels and renal lipid accumulation, but negatively correlated with the eGFR. Conversely, the expression of Sirtuin 1 (Sirt1) was significantly reduced in these patients (Fornoni and Merscher, 2020). Conversely, the expression of Sirtuin 1 (Sirt1) was significantly reduced in these patients. As clinical research on DKD continues to advance from multiple perspectives, the therapeutic advantages of traditional Chinese medicine (TCM) are increasingly being recognized (Liu et al., 2016; Shen et al., 2024; Tang et al., 2021).

The Wenyang Jiedu Tongluo Formula (WYJDTLF) has the effects of warming and tonifying yang qi, detoxifying and clearing turbidity, and promoting blood circulation and unblocking meridians. It consists of 10 g of Anemarrhena asphodeloides, 6 g of Phellodendron amurense, 20 g of Ophiopogon japonicus, 20 g of Scrophularia ningpoensis, 15 g of Codonopsis, 20 g of Rehmannia, 20 g of Morinda, 15 g of Schisandra, 30 g of Smilax, 15 g of Cinnamomum, 3 g of Hirudo, 10 g of Bombyx, and 20 g of Cuscuta. *Huangdi’s Internal Classic* states: “Yang qi is like heaven and the sun; if it loses its proper place, it will shorten one’s lifespan without manifesting its benefits.” Therefore, in treating deficiency-syndrome disorders, the primary focus is on warming and tonifying yang qi to restore its ability to illuminate and warm the body. However, in cases of prolonged illness with deficient yang qi, there is often concomitant accumulation of turbid toxins and blood stasis obstructing the meridians. Therefore, both tonifying and expelling pathogens should be employed simultaneously to invigorate yang qi and eliminate turbidity (*Plain Questions*: On the Generation of Vital Energy and Its Connection to Heaven). First recorded in Lan Shi Mi Cang, the combination of Zhi Mu and Huang Bai produces effects of nourishing yin and lowering fire with yin-yang interdependence. It combines tonification with clearing and descending, and detoxification with yin nourishment (Tian et al., 2019). The entire formula integrates the three methods of warming yang, detoxifying, and unblocking meridians into one, restoring yang qi, clearing turbid toxins, and unblocking meridians, thereby achieving the effects of warming and tonifying yang qi, detoxifying and clearing turbidity, and promoting blood circulation and unblocking meridians. Pharmacological studies have shown that the combination of Anemarrhena asphodeloides and Phellodendron amurense can regulate lipid metabolism by modulating the IRE1α/XBP1s pathway to reduce the expression of SREBP-1c. Maidong polysaccharide extract (MPE) can reduce the secretion of interleukin-1β(IL-1β). Schisandrin B(Sch B) can alleviate epithelial-mesenchymal transition and mitochondrial dysfunction in diabetic nephropathy by inhibiting the Akt pathway and activating the AMPK pathway. Hirudin/liposome complexes can reduce kidney damage by downregulating the expression of TGF-β1 and VEGF in the kidneys.

To elucidate the mechanism by which WYJDTLF modulates lipid metabolism through the JAML/SIRT1 signaling pathway and alleviates diabetic kidney disease (DKD), this study employed a combination of transcriptomic and molecular biology approaches to systematically investigate the therapeutic effects of WYJDTLF. The findings demonstrate that WYJDTLF effectively regulates lipid metabolism, reduces renal lipid deposition, and mitigates DKD progression by targeting the JAML/SIRT1 pathway. This research provides novel insights into the treatment of DKD from the perspective of lipid metabolism.

## 2 Materials and methods

### 2.1 WYJDTLF and compositional analysis

WYJDTLF was procured from the Affiliated Hospital of Changchun University of Chinese Medicine. For sample preparation, 0.5 g of WYJDTLF was precisely weighed and transferred into a 2 mL centrifuge tube. Subsequently, 600 µL of a methanol solution containing 2-chloro-L-phenylalanine (4 ppm, stored at −20°C) was added to the tube. The mixture was oscillated for 30 s to ensure homogeneity, followed by ultrasonication and centrifugation to extract the supernatant. The supernatant was then subjected to liquid chromatography-mass spectrometry (LC-MS) analysis for compositional determination.The chemical composition of WYJDTLF was analyzed using an Agilent 1,200 high-performance liquid chromatography (HPLC) system (Agilent, United States) equipped with a Waters Milford MA column (2.1*150 mm, 1.8 µm). The column temperature was maintained at 40°C with a flow rate of 0.25 mL/min and an injection volume of 2 µL. The detection wavelength was set at 254 nm. For the positive ion mode, the mobile phases consisted of 0.1% formic acid in acetonitrile (B2) and 0.1% formic acid in water (A2). For the negative ion mode, the mobile phases were acetonitrile (B3) and 5 mM ammonium formate in water (A3).

### 2.2 Experimental animals and dosing regimens

Spontaneous type II diabetes mellitus (type II DM) C57BL/Ksj db/db mice (db/db mice), males, 6W°years old, 50, were obtained from Nanjing Junke Biological Co. Ltd., (Laboratory Animal Production Licence No.: SCXK (Su) 2020-009), and 10 male homozygous background non-pathogenic mice (db/m mice). The mice were housed in a specific pathogen-free (SPF) environment under controlled conditions, including an ambient temperature of 24°C ± 2°C, relative humidity of 40%–70%, a 12-h light/dark cycle, and adequate ventilation. After a 2-week acclimatization period, the db/db mice were randomly divided into six groups (n = 6 per group), while the db/m mice served as the blank control group (CON group). The experimental groups were as follows: Model group (MOD group): received 10 mL/kg/d of saline via oral gavage. Positive control group (POS group): administered valsartan (purchased from MCE, Cat. No. CGP 48933) at a dose of 10.29 mg/kg/d in aqueous solution via oral gavage. Low-dose WYJDTLF group (WY-L group): treated with 3.3075 g/kg/d of WYJDTLF in aqueous solution via oral gavage. Medium-dose WYJDTLF group (WY-M group): treated with 6.615 g/kg/d of WYJDTLF in aqueous solution via oral gavage. High-dose WYJDTLF group (WY-H group): treated with 13.23 g/kg/d of WYJDTLF in aqueous solution via oral gavage. Blank control group (CON group): Non-diabetic db/m mice received 10 mL/kg/d of saline via oral gavage. The experimental protocol was approved by the Laboratory Animal Ethics Committee of Changchun University of Chinese Medicine (Approval No. 2024149). All animal experiments were conducted in strict compliance with the ethical guidelines of Changchun University of Traditional Chinese Medicine and 3Rs-principle (Replacement, Reduction, and Refinement).

### 2.3 Detection of blood and urine biochemical parameters and renal histopathological changes

The body weight, kidney weight, and blood glucose levels of the mice were measured and recorded weekly. Biochemical indices, including alanine aminotransferase (ALT), aspartate aminotransferase (AST), albumin (ALB), triglycerides (TG), total cholesterol (TC), blood urea nitrogen (BUN), serum creatinine (Scr), low-density lipoprotein cholesterol (LDL-C), high-density lipoprotein cholesterol (HDL-C), urinary creatinine (Ucr), inflammatory factors such as TNF-αand IL-6 were analyzed using commercially available assay kits (Nanjing Jiancheng Bioengineering Institute). Urinary protein levels were quantified using a urine protein assay kit (Nanjing Jiancheng Bioengineering Institute).Whiskers were clipped using sterile tissue clippers, and blood samples were obtained by retro-orbital venous plexus haematology. And euthanasia of mice using slow intraperitoneal injection of sodium phenobarbital at a dose of 150 mg/kg. One kidney from each mouse was fixed in 4% paraformaldehyde for histological analysis. The kidney tissues were subsequently embedded in paraffin, sectioned, and stained with hematoxylin and eosin (H&E), periodic acid-Schiff (PAS), and Masson’s trichrome to evaluate pathological morphology and structural changes. Immunohistochemistry (IHC) was performed to assess the expression levels of junctional adhesion molecule-like protein (JAML) and podocyte markers, including Nephrin and NPHS2, in the kidney tissues. Additionally, immunofluorescence (IF) staining was conducted to localize JAML expression in the kidney tissues of db/db mice.

### 2.4 Real-time fluorescence quantitative PCR analysis

Total RNA was extracted from 20 mg of homogenized mouse kidney tissue using TRIzol Reagent (Servicebio,Cat.No. G3013) following the manufacturer’s instructions. The concentration and purity of the extracted RNA were measured using a spectrophotometer. Subsequently, RNA was reverse-transcribed into complementary DNA (cDNA) using a reverse transcription kit. Quantitative real-time polymerase chain reaction (qRT-PCR) was performed under the following conditions: 95°C (15s)→60°C (35s)→72°C (25s)for a total of 40 cycles. The threshold cycle (Ct) values were determined using β-actin as the internal reference gene. The relative expression levels of target genes, including collagen type I (COL-I), fibronectin (FN), and junctional adhesion molecule-like protein (JAML), were calculated using the 2^(-ΔΔCt) ^method, where ΔΔCt = (Ct of target gene - Ct of internal reference gene).

### 2.5 Western blotting

Mice kidney tissue samples were homogenized using RIPA lysis buffer supplemented with protease and phosphatase inhibitors (Shanghai Biyuntian Biotechnology Co.,Ltd.). The homogenates were then centrifuged at 12,000 rpm for 15 min at 4°C to isolate the total protein supernatant. Protein concentrations were quantified using a bicinchoninic acid (BCA) protein assay kit (Beijing Soleibao Technology Co., Ltd.), and the protein concentrations of all samples were adjusted to ensure consistency. The protein samples were mixed with 5× reducing sample buffer at a 4:1 ratio and denatured by heating in a boiling water bath for 15 min. Based on the molecular weights of the target proteins, appropriate gel concentrations were selected for sodium dodecyl sulfate-polyacrylamide gel electrophoresis (SDS-PAGE) (lower concentration gels for high molecular weight proteins and higher concentration gels for low molecular weight proteins). Following electrophoresis, the proteins were transferred onto polyvinylidene fluoride (PVDF) membranes (Beijing Soleibao Technology Co., Ltd.). It was closed with containment solution (Shanghai Biyuntian Biotechnology Co., Ltd.) for 3 h at room temperature on a shaker. Then the primary antibodies (for the specific dilution ratio and the source of the manufacturer, please refer to the attachment): ACC1, AMPK, FASN, JAML, mSREBP1, nephrin, P-AMPK, pocodin, SCD1, SIRT1, TNF-α and IL-6 monoclonal antibodies diluted according to the ratio of the instructions were added dropwise and incubated at 4°Covernight. After three washes with TBST, the enzyme-labelled secondary antibody corresponding to the primary antibody was added and incubated at room temperature for 2 h. After another thorough wash, specific protein bands were identified using an enhanced chemiluminescence and imaging system (Tanon Chemiluminescence Imaging Analysis System). The intensity of the bands was analysed semi-quantitatively using ImageJ software (AlphaEaseFC 4.0).

## 3 Results

### 3.1 WYJDTLF ameliorates abnormalities of renal function, liver function, inflammatory indicators and lipid metabolism in db/db mice

**FIGURE 1:**
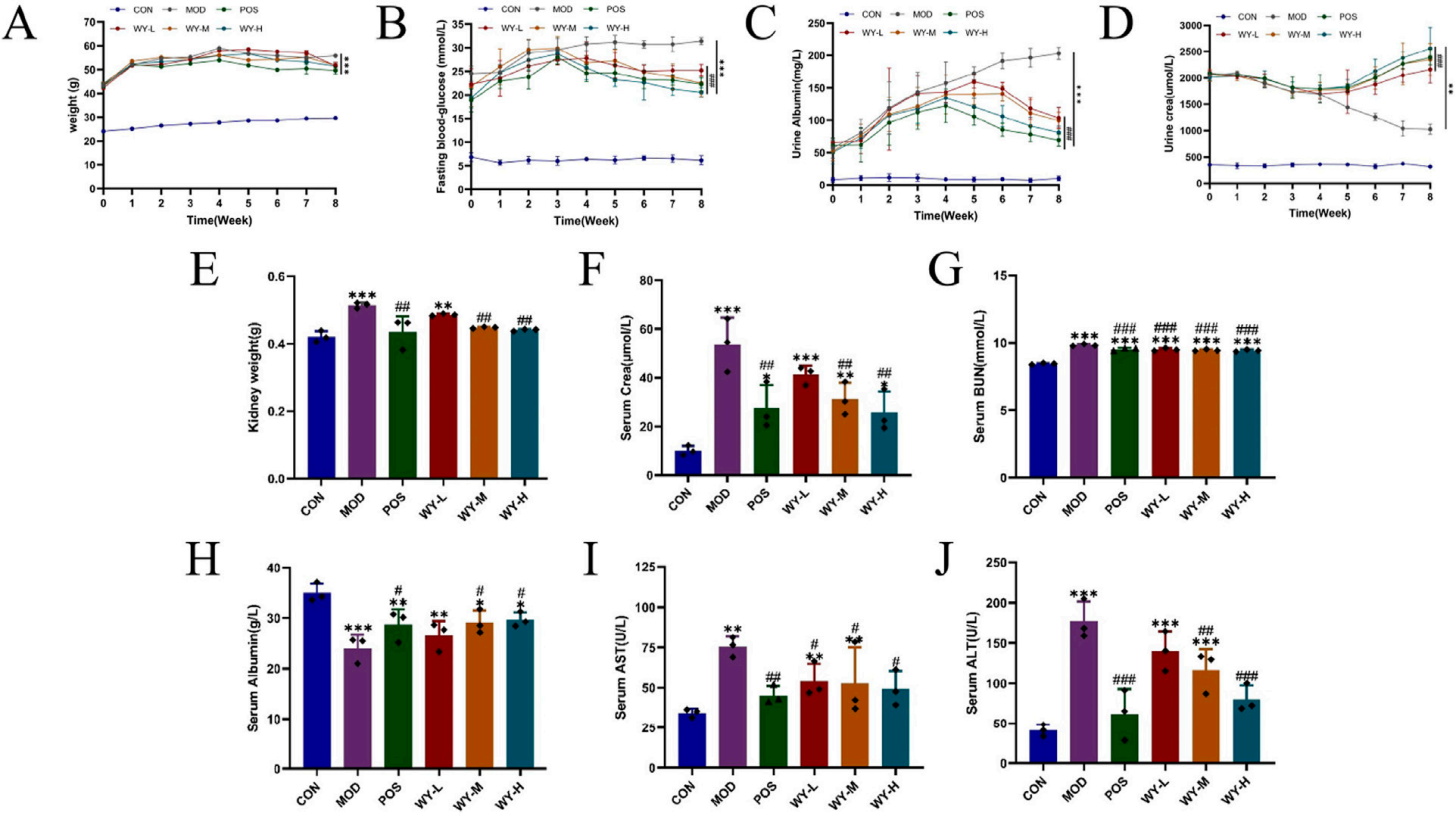
WYJDTLF improves renal function and liver function in db/db mice. The effects of WYJDTLF on the general condition and renal function of db/db mice, including **(A)** body weight, **(B)** blood glucose, **(C)** 24 h-UTP, **(D)** Ucr, **(E)** kidney weight, **(F)** Scr, **(G)** BUN, **(H)** ALB, **(I)** AST, and **(J)** ALT. The data were analysed using analysis of variance and corrected for multiple comparisons, and are expressed as the mean ± standard deviation of 3-6 independent samples. *: compared to control group (Con); #: compared to model group (MOD).

**FIGURE 2:**
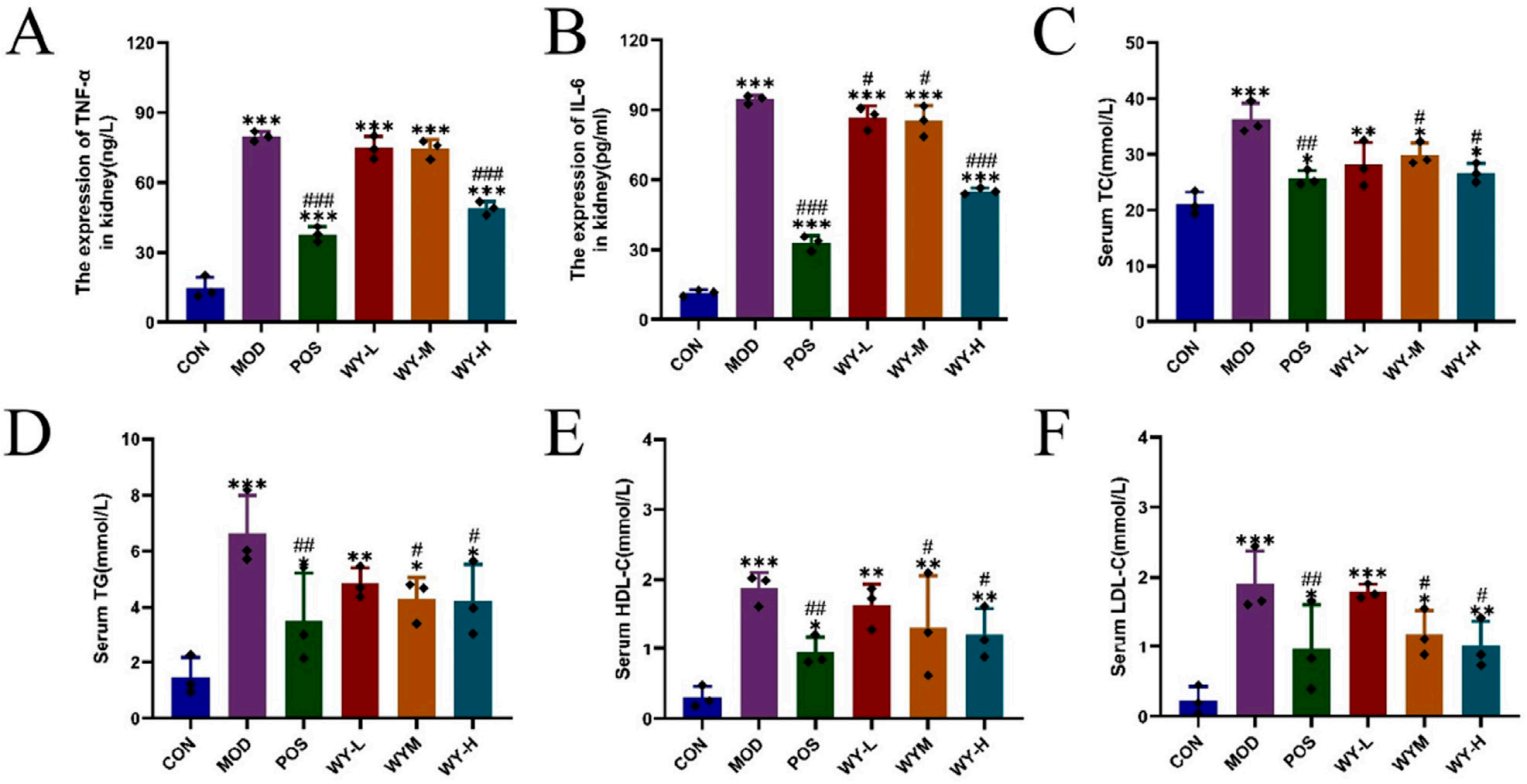
WYJDTLF improves Inflammatory indicators and abnormal lipid metabolism in db/db mice. Effects of WYJDTLF on Inflammatory indicators and abnormal lipid metabolism in mice with **(A)** TNF-α, **(B)** IL-6, **(C)** TC, **(D)** TG, **(E)** HDL-C, and **(F)** LDL-C. The data were analysed using analysis of variance and corrected for multiple comparisons, and are expressed as the mean ± standard deviation of 3-6 independent samples. *: compared to control group (Con); #: compared to model group (MOD).

In this study, we established a diabetic kidney disease (DKD) model using C57BL/Ksj db/db mice, which exhibited characteristic symptoms including depression, bradykinesia, unresponsiveness, weight gain, polyuria, polydipsia, and polyphagia. Following an 8-week treatment, different doses of WYJDTLF could improve renal function indicators such as UACR, BUN, and ALB, as well as liver function indicators such as ALT, AST, and ALB to varying degrees (Figures 1A–J), and could also reduce the activation of inflammatory factors and improve abnormal lipid metabolism (Figures 2A–F).

These findings suggest that WYJDTLF exerts both renal protective effects and positive regulatory effects on lipid metabolism. However, the precise mechanisms underlying these therapeutic effects remain to be elucidated and warrant further investigation.

### 3.2 WYJDTLF amelioration of renal pathological damage in db/db mice

**FIGURE 3:**
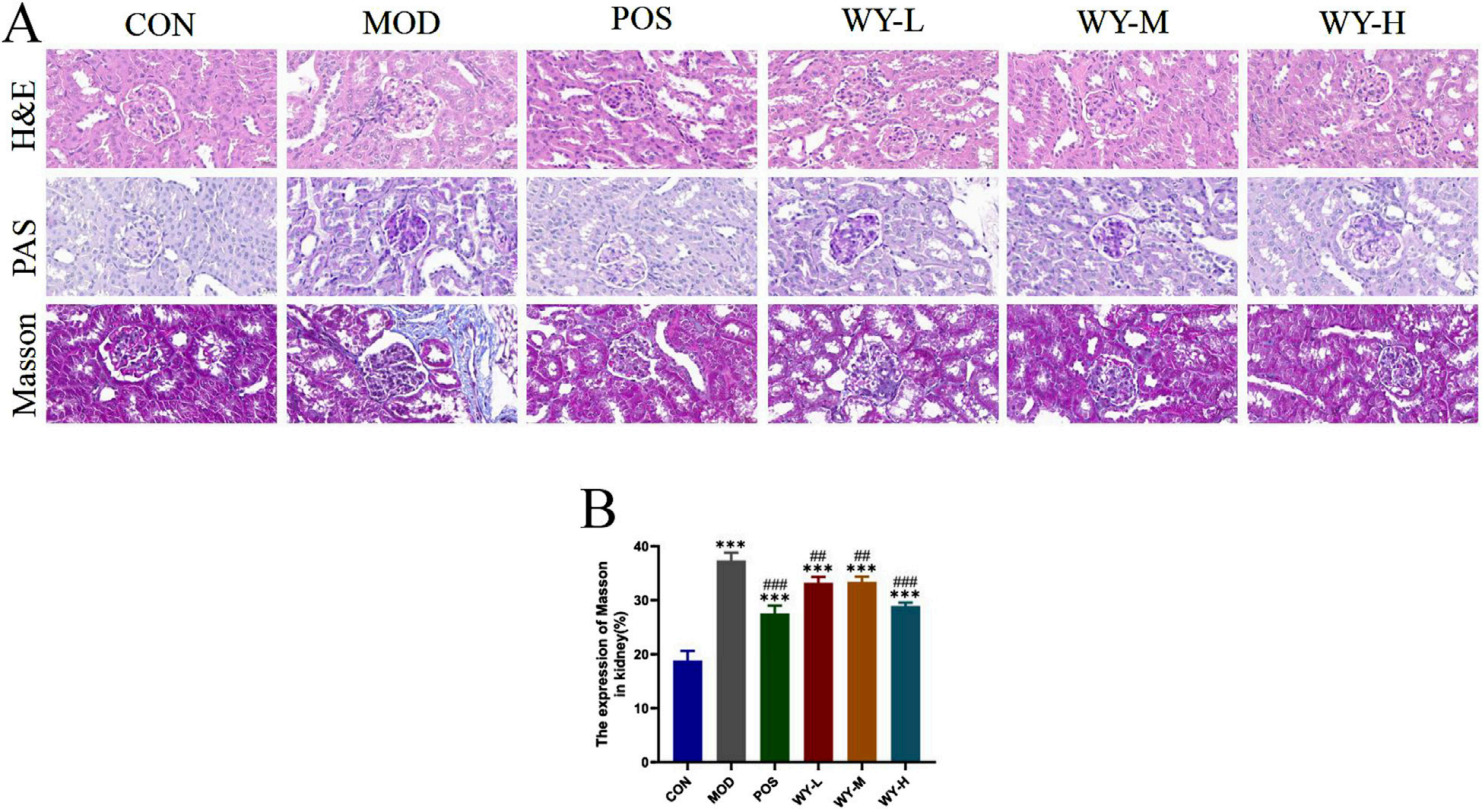
WYJDTLF ameliorates nephropathological injury in the kidneys of db/db mice. **(A)** The degree of renal pathological damage was detected by H&E, PAS and MASSON staining, **(B)** ImageJ software semi-quantitatively analysed the relative area of renal pathological damage under MASSON staining. The data were analysed using analysis of variance and corrected for multiple comparisons, and are expressed as the mean ± standard deviation of 3-6 independent samples. *: compared to control group (Con); #: compared to model group (MOD).

To evaluate the renoprotective effects of WYJDTLF, we conducted comprehensive histopathological analyses using hematoxylin-eosin (H&E), periodic acid-Schiff (PAS), and Masson’s trichrome staining in db/db mice. Histopathological examination revealed characteristic diabetic nephropathy alterations in the MOD group, including glomerular hypertrophy, mesangial matrix expansion, formation of Kimmelstiel-Wilson nodules, microaneurysmal dilatation, and lipid droplet accumulation. Masson’s trichrome staining demonstrated significant collagen deposition and renal fibrosis in the MOD group (Figure 3). Notably, treatment with WYJDTLF or the positive control drug valsartan significantly ameliorated these pathological changes. Both interventions effectively reduced glomerular hypertrophy, attenuated mesangial matrix expansion, and decreased microaneurysmal dilatation. Furthermore, the areas of Kimmelstiel-Wilson nodules, lipid droplet accumulation, and renal fibrosis were markedly reduced in the treatment groups compared to the MOD group. These results suggest that WYJDTLF attenuates pathological injury in the kidneys of db/db mice.

### 3.3 WYJDTLF amelioration of renal fibrosis in db/db mice

**FIGURE 4:**
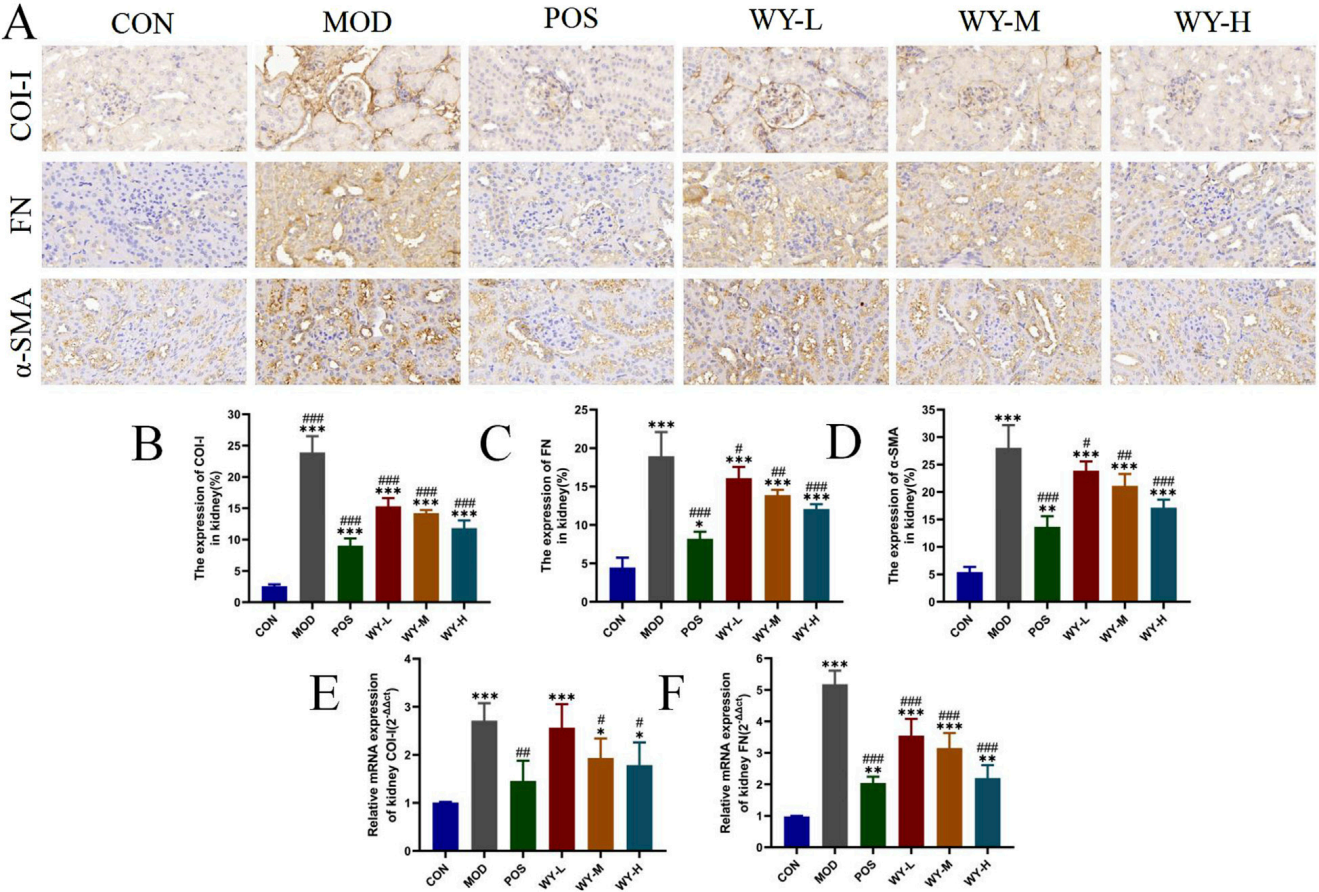
WYJDTLF ameliorates renal fibrosis in db/db mice. **(A)** IHC detected the relative expression of fibrotic proteins COI-I, FN and α-SMA in renal tissues. **(B–F)** PCR to detect the relative mRNA expression of COI-I, FN in renal tissues. ImageJ software semi-quantitatively analysed the relative area of renal fibrosis (blue collagen fibres). The data were analysed using analysis of variance and corrected for multiple comparisons, and are expressed as the mean ± standard deviation of 3-6 independent samples. *: compared to control group (Con); #: compared to model group (MOD).

To further investigate the anti-fibrotic effects of WYJDTLF, we employed immunohistochemistry (IHC) and polymerase chain reaction (PCR) techniques to assess renal fibrosis markers in db/db mice. Both IHC and PCR analyses revealed significant upregulation of key fibrotic proteins, including collagen type I(COL-I), fibronectin (FN), and alpha-smooth muscle actin (α-SMA), in renal tissues of the MOD group, consistent with the observed expansion of fibrotic areas (Figure 4). Therapeutic intervention with either WYJDTLF or the positive control drug valdecoxib resulted in differential downregulation of these fibrotic markers. Both treatments significantly reduced the expression levels of COL-I, FN, and α-SMA, suggesting their potential to inhibit extracellular matrix accumulation and epithelial-mesenchymal transition processes, these results indicated that WYJDTLF could attenuate the degree of renal fibrosis in db/db mice.

### 3.4 WYJDTLF alleviates podocyte damage in db/db mice

**FIGURE 5:**
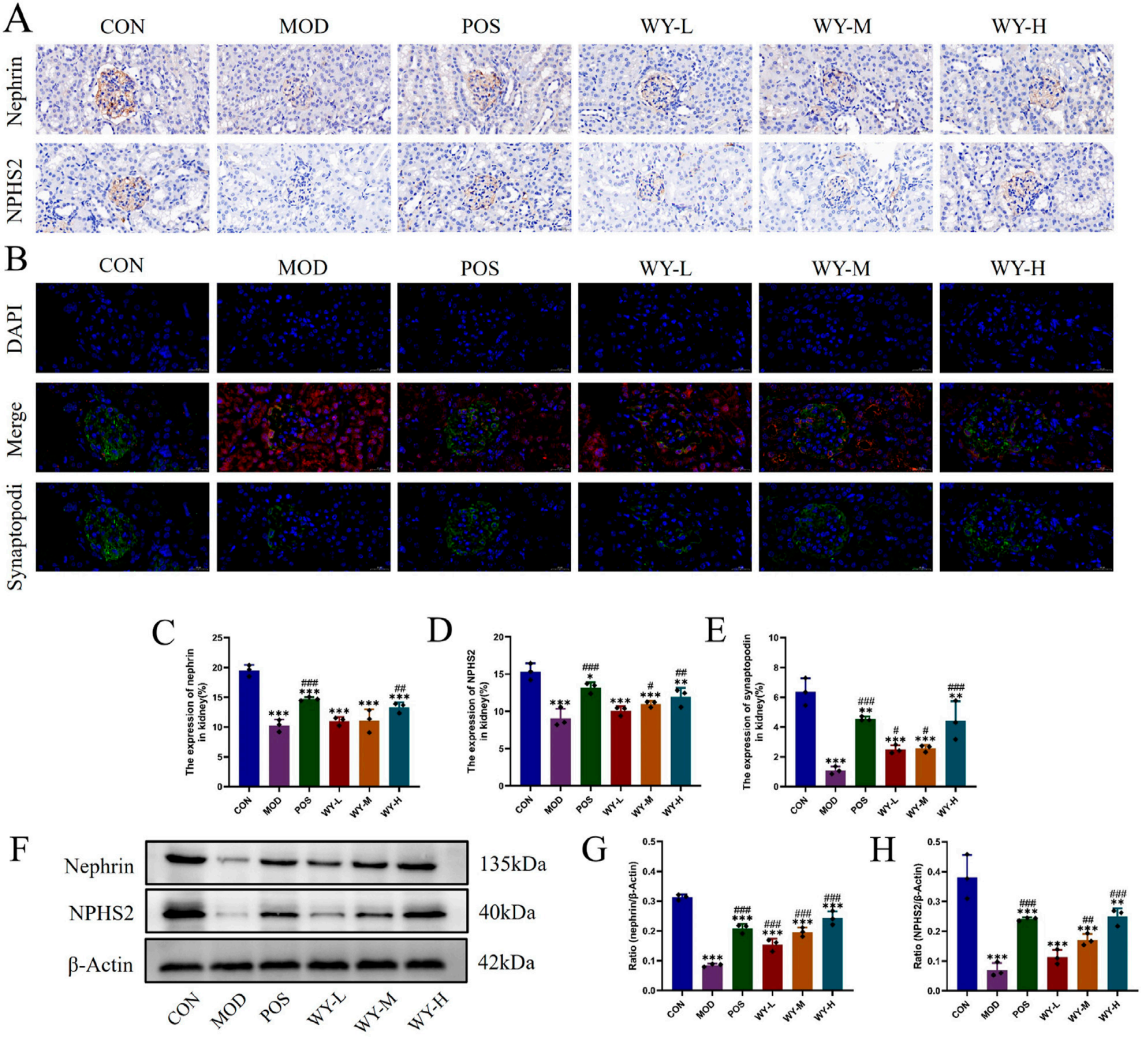
WYJDTLF attenuates podocyte injury. **(A)** IHC detection of the relative expression of two podocyte marker proteins Nephrin and NPHS2 in renal tissues. **(B)** Immunofluorescence detection of podocyte marker protein Synaptopodin in renal tissues, **(C–E)** ImageJ software semi-quantitatively analysed the relative area of Nephrin, NPHS2 and Synaptopodin expression, **(F–H)** WB detection of the relative protein expression of nephrin and pocodin in renal tissues, and ImageJ software for grey scale analysis of the bands. The data were analysed using analysis of variance and corrected for multiple comparisons, and are expressed as the mean ± standard deviation of 3-6 independent samples. *: compared to control group (Con); #: compared to model group (MOD).

To investigate the protective effects of WYJDTLF on podocyte injury, we employed a comprehensive approach utilizing immunohistochemistry (IHC), immunofluorescence, and Western blot (WB) techniques in db/db mice. The IHC and WB analyses revealed significant downregulation of podocyte-specific marker proteins, including Nephrin and NPHS2, in the MOD group compared to the CON group, indicating substantial podocyte damage (Figures 5A,C,D). Immunofluorescence analysis further demonstrated reduced expression of synaptopodin, a critical cytoskeletal protein in podocytes, in the MOD group (Figures 5B,E). WYJDTLF treatment significantly ameliorated these pathological alterations. The formula effectively upregulated Nephrin and NPHS2 expression, reduced mesangial matrix expansion, and attenuated glomerular basement membrane thickening. These findings suggest that WYJDTLF exerts protective effects on podocyte integrity and function, potentially through its multi-target mechanisms that regulate podocyte-specific protein expression and maintain glomerular filtration barrier integrity in diabetic nephropathy.

### 3.5 WYJDTLF regulates the JAML/SIRT1 signalling pathway to improve lipid metabolism

**FIGURE 6:**
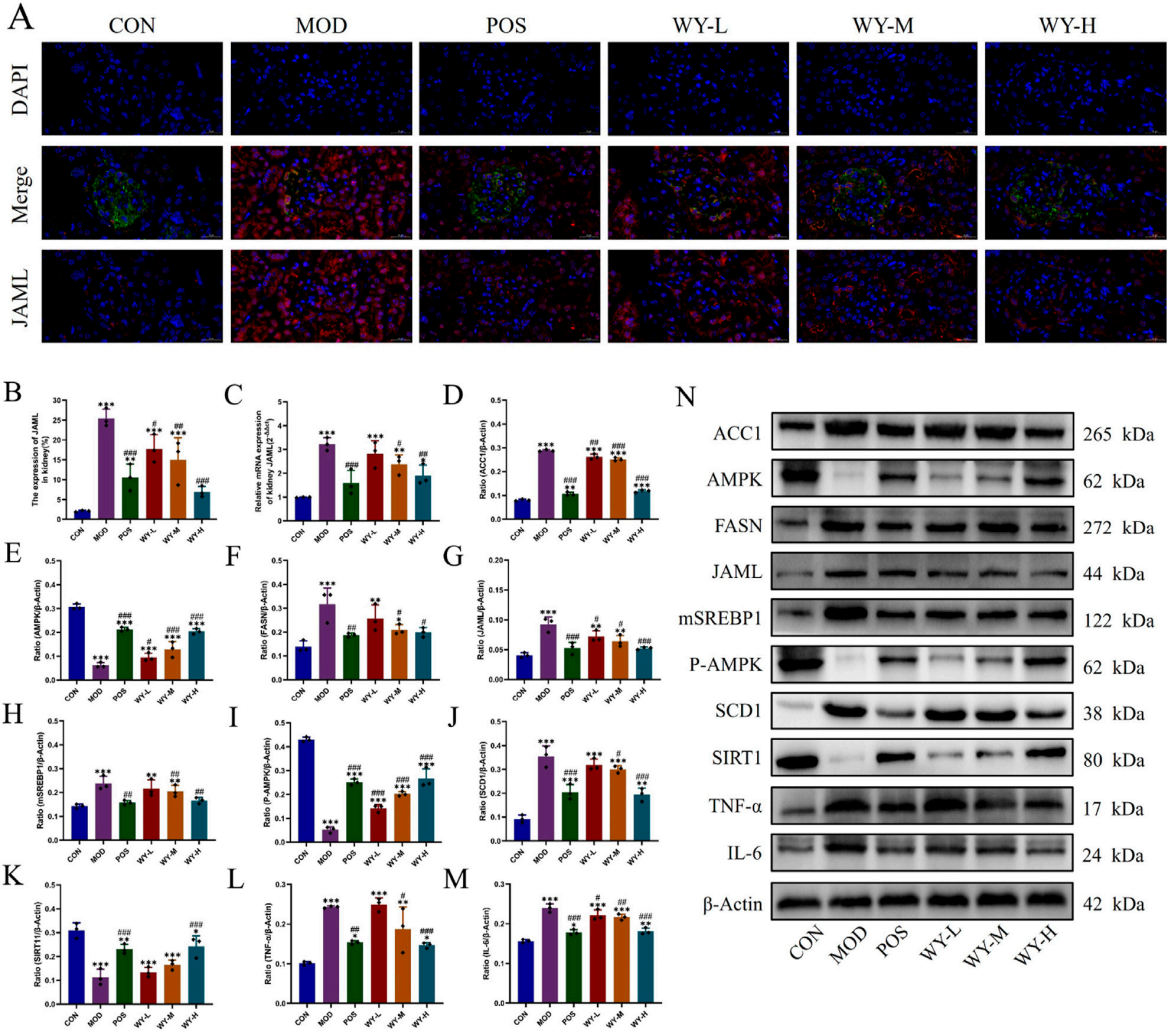
WYJGTLF attenuates podocyte injury by inhibiting the JAML/SIRT1 signalling pathway. **(A–C)** Immunofluorescence was used to detect JAML in renal tissues, PCR was used to detect the relative mRNA expression of JAML in renal tissues, and the relative area of JAML expression was analysed using ImageJ software. **(D–N)** Relative protein expression of ACC1, AMPK, FASN, JAML, mSREBP1, P-AMPK, SCD1, SIRT1, TNF-α and IL-6 in renal tissues was detected by WB, and the bands were analysed in grey scale using ImageJ software. The data were analysed using analysis of variance and corrected for multiple comparisons, and are expressed as the mean ± standard deviation of 3-6 independent samples. *: compared to control group (Con); #: compared to model group (MOD).

We investigated renal lipid deposition and the JAML/SIRT1 signaling pathway in db/db mice using immunofluorescence, PCR, and WB analyses. Immunofluorescence and PCR results demonstrated significantly elevated JAML fluorescence intensity and expression levels in the MOD group compared to the CON group. WYJDTLF treatment effectively reduced mesangial matrix expansion, attenuated glomerular basement membrane thickening, and decreased renal lipid deposition, indicating its potential to ameliorate abnormal lipid metabolism in diabetic nephropathy (Figures 6A–C). WB analysis showed that compared with the CON group, the lipid synthesis markers (ACC1, FASN, JAML, mSREBP1 and SCD1) and inflammatory indicators (TNF-α and IL-6) in the MOD group were significantly upregulated, while AMPK, P-AMPK and SIRT1 were downregulated. Notably, WYJDTLF administration significantly reversed these molecular alterations (Figures 6D–N). These findings suggest that WYJDTLF ameliorates podocyte injury through modulation of the JAML/SIRT1 pathway, potentially by restoring lipid homeostasis and improving cellular energy metabolism in diabetic nephropathy. The multi-target effects of WYJDTLF on lipid metabolism and energy regulation provide novel insights into its renoprotective mechanisms in diabetic kidney disease.

## 4 Discussion

**FIGURE 7:**
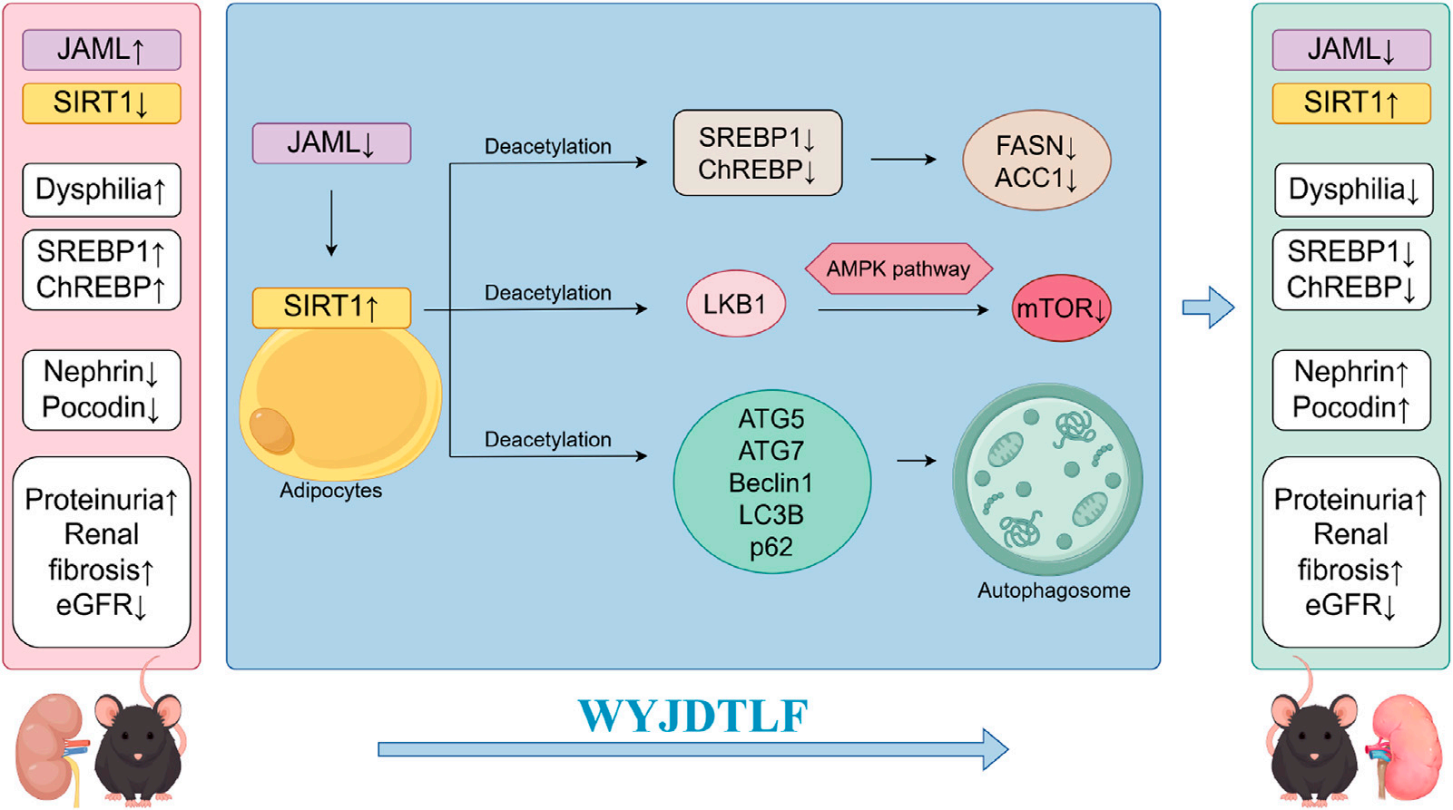
Schematic diagram of the mechanism by which WYJDTLF improves adverse renal dysphilia effects through the JAML/SIRT1 pathway.

This study aimed to elucidate the molecular mechanisms underlying the regulatory effects of WYJDTLF on lipid metabolism in db/db mice, a well-established model of DKD. Our experimental findings demonstrate that WYJDTLF exerts renoprotective effects by ameliorating dysphilia through modulation of the JAML/SIRT1 signaling pathway. These results provide a scientific rationale for the clinical application of WYJDTLF and offer potential therapeutic strategies for DKD management (Figure 7). The induction of oxidative stress, inflammation, fibrosis and apoptosis caused by lipid accumulation and lipid metabolites is known as lipotoxicity (Engin, 2017). The accumulation of toxic lipid metabolites triggers a pathogenic cascade involving inflammatory cell infiltration, pro-inflammatory cytokine release, and activation of fibrotic and apoptotic pathways. This nephrotoxic mechanism, termed as nephrolipotoxicity, represents a crucial pathogenic link between dysphilia and the development of diabetic nephropathy and renal dysfunction (Thongnak et al., 2020). The occurrence of disorders of lipid metabolism is mainly related to lipid synthesis, fatty acid uptake and oxidative imbalance. Emerging evidence indicates that dysregulated lipid metabolism plays a pivotal role in diabetic kidney disease (DKD) progression. Renal histopathological analyses reveal progressive exacerbation of renal fibrosis, lipid accumulation, inflammatory indicators, and JAML/Sirt1 signaling pathway abnormalities correlating with disease severity. These findings suggest that therapeutic modulation of lipid metabolism through JAML/Sirt1 pathway inhibition may represent a promising strategy to mitigate lipid deposition, inflammatory indicators, prevent podocyte injury, and ultimately delay DKD progression (Kang et al., 2015).

Junctional adhesion molecule-like protein (JAML) is a protein localised on the plasma membrane in the region of intercellular contact. As a new member of the JAM family, it plays a key role in immune cell activation, inflammation and lipid metabolism, and is widely found in a variety of intrinsic and adaptive immune cells (Fu et al., 2020; Luissint et al., 2008; Moog-Lutz et al., 2003; Witherden et al., 2010). It has been demonstrated that JAML expression is positively correlated with elevated serum creatinine and lipid metabolism indices and negatively correlated with glomerular filtration rate in patients with DKD (Fornoni and Merscher, 2020). Clinical studies have demonstrated a significant upregulation of JAML expression in renal tissues affected by ischemia-reperfusion injury (IRI), with this elevated state persisting for up to 72 h post-injury. Immunohistochemical analysis revealed prominent JAML expression in renal tubular epithelial cells and macrophages within the tubular interstitium of patients diagnosed with acute tubular necrosis (ATN). These findings suggest that JAML may play a crucial role in the pathogenesis of renal IRI and subsequent tubular injury, potentially serving as a novel biomarker or therapeutic target in acute kidney injury (AKI) management (Huang et al., 2022; Gu et al., 2022). Resveratrol was found to modulate the JAML/Sirt1 lipid synthesis pathway, ameliorate circulating lipid and glucose concentration defects, reduce renal lipid deposition, and ameliorate diabetic kidney damage in mice. Fu (Fu et al., 2020) investigation revealed a novel JAML-SIRT1 signaling axis that regulates lipid metabolism through SREBP1 modulation. Specifically, downregulation of JAML enhanced SIRT1 expression, leading to increased SREBP1 acetylation and subsequent suppression of lipogenic activity. Furthermore, JAML-mediated reduction of SIRT1 expression inhibited AMPK phosphorylation, resulting in decreased SREBP1 phosphorylation and consequent activation of lipid synthesis pathways. These findings demonstrate a dual regulatory mechanism of JAML in lipid metabolism, suggesting its potential role in renal lipotoxicity associated with kidney diseases.

Sirt1 is a member of the silencing information regulator 2 superprotein family (Liu et al., 2021; Zhang et al., 2020), As a highly conserved NAD + dependent deacetylase, its function of sensing the cellular energy state and maintaining its stability is directly related to intracellular NAD + concentration (Ding et al., 2024). Sirt1 is involved in many biological processes such as drug resistance, energy metabolism, cell proliferation, tumour development, autophagy and apoptosis (Kitada et al., 2019; Liang et al., 2008; Michan and Sinclair, 2007; Ou et al., 2014; Packer, 2020). Sirt1 is a positive regulator of autophagy (Yang et al., 2018),and when activated promotes autophagy by deacetylating autophagy-related proteins such as autophagy-related factors (ATG) 5, ATG7, Beclin-1, light chain 3 beta (LC3B) and p62. Furthermore, Sirt1 inhibits ChREBP expression, crosses over with AMPK and mTOR pathways, ameliorates lipid deposition, and regulates energy metabolism (Ding and Choi, 2015; Lu et al., 2024).

In 2020, the concept of the ‘JAML-SIRT1 axis’ was first proposed, and research found that silencing JAML could restore Sirt1 protein levels and AMPKa activity (Fu et al., 2020). Sirt1 regulates the transcription of lipid metabolism-related genes and the post-translational modification of related proteins by deacetylating histones and non-histones, thereby protecting cells from lipotoxicity damage. Additionally, Sirt1 activates the AMPK pathway by deacetylating LKB1 (LyS48) (Shackelford and Shaw, 2009). Furthermore, Sirt1 regulates cellular responses by modulating the activity of numerous enzymes, transcription factors, and cofactors such as SREBP1 and its downstream target genes associated with fatty acid and cholesterol synthesis, thereby altering energy status and regulating lipid metabolism in podocytes (Motonishi et al., 2015). This study reveals the central role of the JAML-SIRT1 axis in abnormal lipid metabolism in podocytes, providing a new perspective for mechanistic research on diabetic nephropathy.

The podocyte, as the fundamental structural component of the glomerular filtration barrier, plays a pivotal role in maintaining renal function. Pathological alterations in podocyte homeostasis, including cellular hypertrophy, foot process effacement, cytoskeletal rearrangement, density reduction, lncRNA dysregulation, and apoptosis, contribute significantly to the development of proteinuria, glomerular filtration rate abnormalities, and elevated serum creatinine levels. Emerging evidence indicates that multiple pathological mechanisms, particularly lipotoxicity, hemodynamic disturbances, oxidative stress, and autophagy dysfunction, collectively contribute to podocyte injury, thereby accelerating the progression of DKD to ESRD (Li et al., 2023). Therefore, further studies to stabilise the function of podocytes by regulating lipid metabolism will be key to slowing down the progression of DKD. Studies have shown that the JAML/Sirt1 signalling pathway has the function of regulating lipid metabolism and plays a regulatory role in the development of DKD (Gu et al., 2022).

Through mass spectrometry analysis, we confirmed that WYJDTLF contains substances such as tyrosol, nicotinic acid and leucine. Tyrosol is a polyphenolic compound with anti-inflammatory properties. It can reduce the expression of inflammatory factors such as IL-1β, TNF-α, and IL-6 by activating the PI3K/Akt pathway, and upregulate Sirt1 expression (Qi et al., 2020). Nicotinic acid is a source of NAD+, a cofactor for Sirt1, while leucine stimulates the AMPK/Sirt1 axis and amplifies the effects of other AMPK/Sirt1-activating compounds. When used in combination, they increase the expression of P-AMPK and Sirt1 in adipocytes and myotubes, thereby reducing lipid accumulation (Bruckbauer et al., 2017). These study confirms that WYJDTLF can alleviate foot cell damage by regulating lipid metabolism through multiple targets. Its multi-component synergistic action provides a potential strategy for intervening in kidney damage related to dysphilia.Using methods such as PCR,Western blot analysis, and immunofluorescence, we validated that WYJDTLF can significantly inhibit the JAML/SIRT1 signalling pathway, thereby alleviating inflammatory responses, reducing the production of JAML and mSREBP1 that lead to lipid deposition, and promoting the production of SIRT1, which counteracts lipid deposition. This results in increased expression levels of podocyte gap junction protein Nephrin, NPHS2, and reduce podocyte structural damage, thereby protecting against kidney damage in diabetic nephropathy. This study first revealed the molecular mechanism by which WYJDTLF improves renal dysphilia through the JAML/SIRT1 pathway.

The adult dosage of WYJDTLF is 150 mL per dose. It has been extensively used in clinical practice for over 10 years and has been validated through basic research, demonstrating good efficacy and safety (Dong et al., 2010; Jin et al., 2025; Li et al., 2025). However, several limitations should be acknowledged in this study. First, our study only explored the mechanism of WYJDTLF in regulating lipid metabolism at the *in vivo* animal experiment level. However, further validation of the specific regulatory mechanism of the JAML/Sirt1 pathway through gene knockout experiments, as well as supplementary cell experiments and clinical trials, is essential to provide more robust theoretical support for clinical translation. This area remains unexplored in this study, and the exploration of the mechanism is not sufficiently detailed or in-depth. Second, podocyte injury involves multifaceted pathological processes beyond lipid metabolism, including autophagy dysregulation, oxidative stress, immunoinflammatory responses, cellular pyroptosis, hemodynamic disturbances, mitochondrial dysfunction, and lncRNA abnormalities, which warrant further investigation to fully understand the therapeutic potential of WYJDTLF in DKD management.

## Data Availability

The original contributions presented in the study are included in the article/supplementary material, further inquiries can be directed to the corresponding authors.
